# Probiotic administration among free-living older adults: a double blinded, randomized, placebo-controlled clinical trial

**DOI:** 10.1186/s12937-016-0198-1

**Published:** 2016-09-10

**Authors:** Lina Östlund-Lagerström, Annica Kihlgren, Dirk Repsilber, Bengt Björkstén, Robert J. Brummer, Ida Schoultz

**Affiliations:** 1 Nutrition and Physical Activity Research Centre, Örebro University, Örebro, Sweden; 2 Nutrition Gut Brain Interactions Research Centre, School of Health and Medical Sciences, Faculty of Health and Medicine, Örebro University, Örebro, Sweden; 3 Institute of Environmental Medicine, Karolinska institutet, Stockholm, Sweden

**Keywords:** Older adults, Digestive health, Wellbeing, Lactobacillus reuteri, Probiotics

## Abstract

**Background:**

Diseases of the digestive system have been found to contribute to a higher symptom burden in older adults. Thus, therapeutic strategies able to treat gastrointestinal discomfort might impact the overall health status and help older adults to increase their overall health status and optimal functionality.

**Objective:**

The aim of this double-blinded, randomized, placebo-controlled clinical trial was to evaluate the effect of the probiotic strain *Lactobacillus reuteri* on digestive health and wellbeing in older adults.

**Methods:**

The study enrolled general older adults (>65 years). After eligibility screening qualified subjects (*n* = 290) participated in a 2-arm study design, with each arm consisting of 12 weeks of intervention of either active or placebo product. Primary outcome measure was set to changes in gastrointestinal symptoms and secondary outcome measures were changes in level of wellbeing, anxiety and stress. Follow up was performed at 8 and 12 weeks.

**Results:**

No persistent significant effects were observed on the primary or secondary outcome parameters of the study. A modest effect was observed in the probiotic arm, were levels of stress decreased at week 8 and 12. Similarly, we found that subjects suffering from indigestion and abdominal pain, respectively, showed a significant decrease of anxiety at week 8 after probiotic treatment, but not at week 12.

**Conclusion:**

The RCT failed to show any improvement in digestive health after daily intake of a probiotic supplement containing *L. reuteri*. Neither was any significant improvement in wellbeing, stress or anxiety observed. Even though the RCT had a negative outcome, the study highlights issues important to take into consideration when designing trials among older adults.

**Trial registration:**

Clinicaltrials.gov/NCT01837940.

## Introduction

The aging process is characterized by loss of function; particularly cognitive functions, as well as gastrointestinal problems are widespread phenomena among older adults. Diseases of the digestive system have been found to contribute to a higher symptom burden in a population of Swedish free-living older adults [1]. Acute and chronic diarrhea is frequently observed often resulting in an aberrant stool pattern restricting the older adults’ everyday life [1, 2]. A well functioning gastrointestinal tract is essential for older adults to experience life-satisfaction and meaningfulness [3, 4]. Today, little is known about the mechanisms behind age-associated gut problems and few treatment options designed only for older adults exist. Hence, therapeutic strategies able to treat gastrointestinal discomfort might impact the overall health status and personal integrity among the oldest old.

The diversity of the gut microbiota has been identified as a major contributor to gut health and is known to influence the intestinal barrier function (e.g. the capacity of the intestine to act as a wall against bacterial products and food antigens and yet being able to take up nutrients), as well as being vital for a well-functioning immune system [5, 6]. Moreover, the gut microbiota has recently been identified as an important player in the gut-brain axis, linking emotional and cognitive centers of the brain with peripheral intestinal functions [7]. Therapies able to beneficially affect the gut microbiota composition, such as probiotic bacteria (e.g. live bacterial strains expressing beneficial health effects on the host), might alleviate gastrointestinal discomfort and increase overall health status among older adults.

The probiotic species *L. reuteri* is an endogenous organism [8, 9] able to modulate the gut microbiota through the production of reuterin, a potent antibacterial compound, capable of inhibiting a wide spectrum of microorganisms [10]. *L. reuteri* has been identified as a promising therapy in various gastrointestinal disorders [11–13], including functional chronic constipation [14, 15]. Here, we aim to conduct a double-blinded, randomized, placebo-controlled study with the aim to investigate digestive health after the intake of a dietary supplement, consisting of the probiotic strain *L. reuteri*, during a three-month period. Furthermore, we evaluated whether a change in digestive health correlated to an improved subjective perception of wellbeing, and lower levels of depression, anxiety and stress.

## Methods

### Study participants

The study population was recruited through advertisements in local and regional newspapers. At senior living homes information meetings were held and flyers were handed out to all residents. The study population was recruited from the region of Örebro, within a 45 km radius from the City center. Residents at the senior living homes were invited to participate after careful presentation of the protocol, individual discussion if needed, and signing of the consent form. A total population of 307 eligible free-living older adults (mean age 73.1, range 65-98) was recruited between January and March 2013. All participants lived in their own home. Each study participant was assigned a contact person (a medical student informed in detail about the study procedures).

### Inclusion and exclusion criteria

Each older adult had to meet the following criteria to be enrolled in the study:

- Informed consent signed by study participant
- Age ≥65 years
- Mentally and physically fit to complete questionnaires during the study period.

Older adults meeting any of the following criteria were excluded from the study:

- Any known gastrointestinal disease, with strictures, malignance’s and ischemia.
- Inflammatory bowel diseases
- Participation in other clinical trials in the past three months.

### Study design

This study was a double-blinded, randomized, placebo-controlled clinical trial enrolling free-living older adults (≥65 years) representing the general population in the region of Örebro, Sweden. The treatment period was 12 weeks and the intervention parameters were evaluated at week 0, 8 and 12. All subjects entered the intervention phase within two weeks. Two weeks prior follow up a reminder was sent by mail to all participants with a suggestion of day and time to meet or phone their contact person (assigned at enrolment). The participants were asked to confirm the time one-week prior follow up. Three phone calls were placed on three consecutive days to participants not responding to the letter. As a final step a home visit was made on the day assigned for follow up. The contact person assisted the study participant to assure that the questionnaires were answered correctly. A study diary was provided to all participants were the daily stool frequency was monitored and new medications were reported. The active study product consisted of a stick-pack containing freeze-dried *L. reuteri* DSM 17938 (1 × 10^8^ colony forming units/stick-pack), rhamnose, galactooligosaccharide and maltodextrin to a total weight of 1 g. The placebo product consisted of maltodextrin with the same appearance, color and taste as the active study product, identically packaged and stored. The study product was packed and labeled by BioGaia AB. Active and placebo stick-packs were packed identically with the number of the subject and storing instructions. All products were blinded and randomized by BioGaia AB. For the research team, a blinded randomization list was provided with the subject sequence and subject number. The research team allocated the study participants by randomly assigning the pre-labeled study product to the participants and a screening record in which all screened subjects were registered. For each subject enrolled, date of enrolment and ID number of the trial subject was recorded. The identity of the specific product was blinded to all subjects, contact persons and investigators. The coding was only known to the representative of the BioGaia AB responsible for product randomization and had no other involvement in the trial. All other personnel at Bio Gaia AB involved in the study as well as the research team and study staff remained blinded to the study treatment until completion of the statistical analysis and discussion of the findings.

A statistical power calculation was performed on GSRS data from the first 100 participants indicating that 74.2 % of older adults suffer from gastrointestinal discomfort (judged by a score of >2 at any domain of GSRS). A sample size of 150 per group (300 in total) provided 80 % power with 95 % confidence interval to detect a minimum clinical improvement in gastrointestinal discomfort of 20 % among the intervention arm and 5 % of the placebo arm. Based on these calculations a decision was made to aim to include at least 200 subjects in each group, 400 in total, to assure that the study was adequately powered. In total 307 study participants were recruited of these 290 were enrolled and randomized (Fig. 2). The study participants were instructed to take two stick-packs per day, at breakfast or lunch, for a 12 week-period and to maintain their normal dietary habits during the study-period.

## Data collection

### Demographic data

The following demographic information was collected in order to better define the study population: age, sex, education level, marital status, retirement age and smoking habits (Table 1).

**Table 1:** Baseline characteristics

Characteristics	Probiotic group	Placebo group
Sex	125	124
Female %	57	65.6
Male %	43	34.4
Age (mean (SD))	72.6 (5.8)	72 (5.6)
Marital status %		
Married	51.6	63.6
Widow/Widower	18	23.9
Divorced	18.9	82.6
Single	7.4	4.1
Missing		
Education %		
Lower	47.9	58.2
Higher	42.1	41.8
Missing	0.1	0.1
Retirement age (mean (SD))	63.3 (3.8)	64.4 (3.3)
Smoking		
Yes	2.5	5.7
No	95	94.3
Missing	2.5	0.8
Prescriptions drugs %		
Non-Steroidal Anti-Inflammatory Substances	5	6.6
Blood Pressure Lowering Substances	32.8	27.9
Proton Pump Inhibitors	16.5	11.5
Opiates	3.3	1.6
Over-the-counter drugs %		
GI Motility Modulating Substances	12.9	15.5

### Medications

All prescribed medications taken during the 6 months preceding the study start was assessed by self-reporting by each recruited subject. Current prescription of medications was documented in the study CRF. The distribution of prescribed medications is shown in Table 1.

### Intervention parameters

#### Gastrointestinal symptoms rating scale (GSRS)

Is a validated instrument, [16–18] previously used to assess gastrointestinal discomfort among older adults [19]. The scale measures 15 symptoms, which are divided into 5 domains (i.e. reflux, abdominal pain, indigestion, diarrhea and constipation). The total GSRS score was used, as an estimate of the overall gastrointestinal discomfort.

#### Hospital anxiety and depression scale (HADS)

Is a widely used validated instrument for the evaluation of psychological distress in medical settings as well as in older adults [20, 21]. The instrument consists of 14 items, consisting of two subscales for assessment of anxiety or depression. The total score is used as a measure of psychological distress.

#### EQ-5D-5L (EQ-5D index and EQ-VAS)

EuroQol is a standardized measure of health status [22] and has been validated in older adults [23]. The tool consists of two parts; 5Q-5D, which includes 5 items related to wellbeing and function (mobility, self-care, usual activities, pain/discomfort and anxiety/depression) and the visual analogue scale, 5Q-5D-VAS, ranging from 0 to 100.

#### Perceived stress scale (PSS)

The instrument assess the perception of stress and has been validated among older adults [24, 25]. The scale consists of 10 items, including a number of direct queries about current levels of experienced stress. The respondent answers how often a certain emotion has been present during the past month. A total score was used to illustrate the study participants’ perception of stress.

#### Daily stool frequency

The number of stools per day was monitored in the study diary.

### Monitoring of compliance and adverse events

**Fig. 1:**
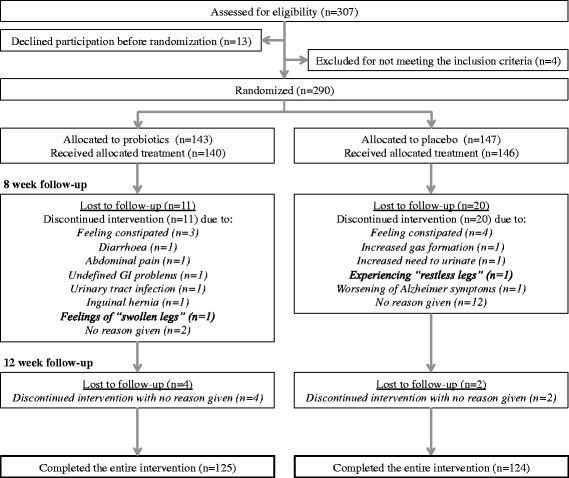
Participant flow and adverse events

Adherence was measured through a study diary that was completed daily and where all study participants marked if they taken the supplement at breakfast or at lunch. Participants overlooking to take the supplement one day per week (12 non-continuous days) or during four consecutive days throughout the study were considered non-compliant, as taking the supplement sporadically could affect the primary outcome. All adverse events occurring after any administration of the study product was followed until the end of the study. All adverse events are shown in Fig. 1. No serious adverse events (e.g. events resulting in death, immediately life-threatening, requiring or prolonging hospitalization or resulting in persistent or significant disability/incapacity) were reported during the study.

## Statistical analyses

### Primary outcome measures

As a first step all data was included in an intention-to-treat (ITT) analysis. In a next step we performed a per-protocol analysis. A responder was considered a subject compliant to the protocol and experiencing a relief in symptoms by a score of 0.5 (the minimal clinical relevant change) on the mean value of GSRS or on any of the domains. Gastrointestinal motility modulating substances (GMMS) and proton pump inhibitors (PPI) were considered to affect this end point analysis. Hence, study subjects taken these medications were excluded from further analysis (*n* = 71). All substances excluded were classified according to the Swedish environmental classification of pharmaceuticals at www.fass.se. As a next step the median (+ IQR) and mean (± SD) score of the GSRS questionnaire for all subjects, including non-compliant participants (*n* = 178), were calculated for week 8 and 12, and are shown as changes from baseline in the results section. Gastrointestinal problems at baseline were defined as a score of >2 at any domain of GSRS, including mild to severe problems. Separate GSRS domain scores for 8 and for 12 weeks were tested for differences between the study arms using a student’s *t*-test or Mann-Whitney *U*-test, (Table 2). Sub analyses were performed in order to identify if participants experiencing gastrointestinal symptoms, defined as a score <2 on any domain of GSRS, experienced any relief in anxiety, depression, stress or wellbeing after probiotic supplement. Eighteen questionnaire items (GSRS) had to be imputed due to missing data. The missing value was replaced with the arithmetic mean of the completed items in the questionnaire, in accordance with the instructions for the questionnaire.

**Table 2:** Statistical findings shown as change from baseline at week 8 and 12

	+8 week follow-up						+12 week follow-up					
	Probiotic		Placebo		ST		Probiotic		Placebo		ST	
					*p*-value						p-value	
Outcome parameter	x̅	Med	x̅	Med	tT	UT	x̅	Med	x̅	Med	tT	UT
GSRS	−0.04 ± 0.6	0 (−0.3–0.3)	−0.08 ± 0.6	−0.1 (−0.30.1)	0.63	0.18	−0.07 ± 0.6	0 (−0.4–0.2)	−0.08 ± 0.6	−0.1 (−0.4–0.2)	0.91	0.42
Diarrhea	−0.09 ± 0.7	0 (−0.7–0.3)	−0.05 ± 1.0	0 (−0.3–0.3)	0.63	0.89	−0.12 ± 0.9	0 (−0.3–0.3)	−0.05 ± 0.9	0 (−0.3–0)	0.61	0.52
Indigestion	−0.14 ± 0.9	−0.3 (−0.6–0.3)	−0.15 ± 0.8	0 (−0.5–0)	0.95	0.82	−0.14 ± 0.9	−0.1 (−0.5–0.3)	−0.13 ± 0.7	0 (−0.5–0.3)	0.94	0.89
Constipation	0.02 ± 1.1	0 (−0.7–0.7)	−0.03 ± 1.0	0 (−0.3–0.3)	0.76	0.46	−0.06 ± 1.1	0 (−0.7–0.3)	−0.10 ± 1.1	0 (−0.3−0.3)	0.82	0.99
Abdom. pain	−0.07 ± 0.6	0 (−0.3–0.2)	−0.07 ± 0.7	0 (−0.3–0.3)	0.94	0.92	−0.08 ± 0.7	0 (−0.3–0.3)	−0.09 ± 0.7	0 (−0.3–0.3)	0.95	0.86
Reflux	0.12 ± 0.7	0 (0–0)	−0.13 ± 0.6	0 (0–0)	0.02^a^	0.02^a^	−0.02 ± 0.7	0 (0–0)	−0.09 ± 0.5	0 (0–0)	0.45	0.33
EQ-5D												
Index	0 ± 0.1	0 (0–0)	−0.01 ± 0.1	0 (−0.1–0)	0.35	0.23	0 ± 0.1	0 (0–0)	0 ± 0.1	0 (0–0)	0.87	0.79
VAS	1.89 ± 10.7	0 (−5–5)	0.60 ± 11.5	0 (−5–5)	0.44	0.76	2.88 ± 9.8	0 (−30–0)	3.47 ± 7.7	5 (0–10)	0.66	0.29
HADS	−0.32 ± 3.5	0 (−2–1)	−0.41 ± 2.9	0 (−2–1.3)	0.86	0.45	−0.18 ± 4.4	−0.5 (−2–1)	0.05 ± 5.2	0 (−2–2)	0.77	0.74
Depression	−0.01 ± 2.1	0 (−1–1)	−0.18 ± 1.9	0 (−1–0)	0.60	0.79	0.04 ± 2.2	0 (−1–1)	−0.01 ± 2.8	0 (−1–1)	0.89	0.91
Anxiety	−0.31 ± 2.1	0 (−1.3–1)	−0.23 ± 1.9	0 (−1–1)	0.79	0.54	−0.22 ± 2.7	0 (−2–0.13)	0.06 ± 3.0	0 (−1–1)	0.55	0.42
PSS	−0.41 ± 4.9	−1 (−3–2)	−0.24 ± 5.4	0 (−3.8–3)	0.82	0.59	−1.55 ± 4.8	−1 (−5–2)	−1.12 ± 4.4	−1 (−4–1.8)	0.57	0.58

x̅ = Mean ± SD

*ST* Significance testing between the two treatment groups

*tT t*-test

*UT* Mann-Whitney *U*-test

*Med* Median (IQR)

^a^significant at the 95 confidence level

### Secondary outcome measures

The analyses of the secondary outcome parameters were performed similar to the primary outcome parameters. Initially, an ITT was performed followed by a per-protocol analysis including a responder analysis of the compliant subjects. As for the primary outcome no significant results between the treatment groups were detected. As a final step a Student’s *t*-test or Mann Whitney *U*-test was performed to analyze the mean and median score, respectively, between the two study arms for the secondary questionnaires (e.g. HADS, PSS, EQ-5D-5L). The two study arms were compared at week 8 and 12 after subtraction of the baseline score.

All analysis was performed blinded. 28 individual questionnaires sheets (HADS: 11, PSS: 17, EuroQol: 0) had to be excluded from the analysis due to missing values in proportions that did not allow for imputation. All data were stored in a common database and statistically analyzed using r version 3.0 (Auckland, New Zeeland). A *p* value < 0.05 was considered to be statistically significant. All reported *p*-values are descriptive, without multiplicity correction.

## Results

### Participant flow and baseline characteristics

Overall 290 older adults were randomized (93 % of 307 eligible study participants; 140 probiotic, 146 placebo treatment, respectively) (Fig. 1). Retention to primary outcome was 85 % (249 analyzed; 125 probiotic, 124 placebo treatment, respectively); 37 subjects were excluded due to loss to follow up (Fig. 1). The baseline characteristics did not differ significantly between the two treatment groups, except that significantly more women and a higher number of divorced study participants was included in the placebo arm (Table 1).

## Primary outcome

**Fig. 2:**
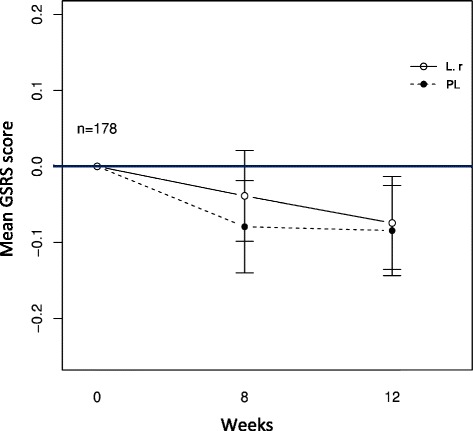
Changes in the primary outcome parameter over the study period. The figure shows change from baseline in GSRS total mean score in the two treatment groups, throughout the intervention period. 178 subjects were included in the analysis, excluding participants taking PPI and GMMS during the study. (Prob) indicates the probiotic group receiving treatment with *L. reuteri* (*n* = 86) and (PL) indicates the placebo group (*n* = 92)

The ITT analysis as well as the per-protocol analysis did not show any significant differences between the two treatment groups (data not shown). The study population reported a mean GSRS score of 1.9 ± 0.8, suggesting that a large proportion of participants suffered from mild gut problems. As no significant differences between the groups could be observed, the trends are presented as mean ± SD in the results section, to illustrate the subtle changes observed throughout the study. Table 2 shows and compares all primary and secondary outcomes (mean and median values) at 8 and 12 weeks. Overall the two groups were similar on almost all outcomes. The total mean score of GSRS did not change markedly in either the placebo or probiotic arm during the study (Fig. 2). Similarly, no significant differences were observed in the separate domains measured by GSRS (Table 2), except for the reflux domain where a significant difference between groups occurred at week 8; the reflux symptoms decreased in the placebo group while increasing in the probiotic group (*p* = 0.02). This trend, however, did not persist at week 12.

## Secondary outcomes

### Hospital and anxiety and depression scale

The two groups scored almost similar at week 8 and 12 and no significant differences could be observed in anxiety or depression score (Table 2). Probiotic treatment did not significantly affect depression score. Neither was probiotic treatment or placebo found to decrease symptoms of anxiety or depression in study participants experiencing problems in these domains at baseline (data not shown). Next, we investigated if study participants suffering from gastrointestinal problems, (as judged by a score >2 on any of the domains of GSRS) did experience any relief in anxiety or depression during the study. For subjects suffering from indigestion symptoms, anxiety decreased by 0.73 ± 1.8 at week 8 in the probiotic arm compared to the placebo group where the symptoms increased by a score of 0.18 ± 1.5, *p* = 0.025. Subjects suffering from abdominal pain showed a similar trend; symptoms of anxiety decreased with 0.75 ± 2.0 during probiotic treatment at week 8, compared to the placebo group where the symptoms increased by a score of 0.4 ± 1.7 (*p* = 0.01). These effects, however, did not persist at week 12. Thus, the observations were not consistent or relevant indicating that neither probiotic or placebo treatment was found to be able to decrease symptoms of depression among participants suffering from gastrointestinal symptoms.

### Perceived stress scale

**Fig. 3:**
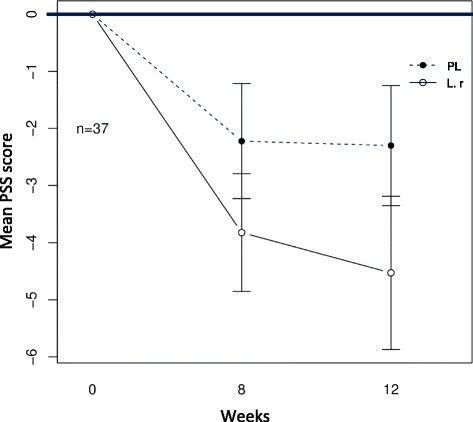
Changes in psychological distress for subjects with elevated stress at baseline. The figure shows the 37 subjects with baseline PSS score ≥15, (Prob) indicates the probiotic group receiving treatment with *L. reuteri* (*n* = 17) and (PL) indicates the placebo group (*n* = 20). A modest effect was found for the probiotic treatment to decrease symptoms of stress over the study period (4.53 ± 4.25 compared to 2.2 ± 4.5 for placebo treatment, respectively (*p* = 0.20, statistical test: Mann-Whitney *U*-test))

No significant differences in stress levels were observed between the two study arms at week 8 and 12, respectively (Table 2). An arbitrary cut off (≥ 15) was chosen in order to investigate whether subjects suffering from elevated stress at baseline changed their PSS scores over the study period. This cut-off value was chosen based on the frequency of stress problems among the study population. A non-significant trend was found for the probiotic treatment to decrease symptoms of stress with (4.53 ± 4.2 compared to 2.2 ± 4.5 for placebo treatment, respectively) over the study period, at week 12 (*p* = 0.20) (Fig. 3). We further investigated whether a significant change in stress level could be observed among study participants experiencing gastrointestinal discomfort at baseline. No such difference was observed (data not shown).

### EQ-5D-5L

Similarly, as for the other outcomes, no significant differences in subjective wellbeing were observed between the probiotic and placebo arm (Table 2). The EQ-VAS and 5Q-5D-index scores were almost similar in both study arms at week 8 and 12, respectively. Both probiotic and placebo treatment was associated with a significantly increased subjective wellbeing at the week 12 (2.88 ± 9.8 and 3.47 ± 7.7 for probiotic and placebo treatment, respectively). Neither did we identify any differences in wellbeing between probiotic and placebo treatment in subjects suffering from gastrointestinal symptoms at baseline (data not shown).

### Daily stool frequency

Probiotic treatment did not influence the stool frequency in the outlined study. No significant differences were found between the probiotic and placebo arm. Nor could a difference bee seen within each group (data not shown).

## Adherence and adverse events

Two hundred and forty nine study participants of the 290 that were enrolled finished the RCT.

Fifteen study participants in the probiotic arm and 22 participants in the placebo arm were lost to follow up during week 8 and 12 (Fig. 1). Participants reporting the reason for discontinuation as a result of illness were equally distributed between the two treatment groups.

## Discussion

In the present study, the effect of *L reuteri* DSM17938 was investigated on digestive health, wellbeing and psychological distress in older adults in a randomized, placebo-controlled clinical trial. DSM17938 is a well-studied probiotic strain and has been found to exert a positive effect on constipation and diarrhea in adults and are currently used as treatment of colic in infants in Sweden [11, 15, 26, 27]. To our knowledge this is the first study investigating the effect of *L reuteri* in free-living older adults. However, 12 weeks of treatment failed to show a difference in any of the outcome measures.

The study aimed to include a population of older adults representing the general population. This is an important group to investigate in a healthy aging perspective, as these older adults in the near future will be in need of elevated health care. Thus, it is within this group it is important to perform interventions that will promote health and optimal functionality through out life.

The study population reported a mean GSRS score of 1.9 ± 0.8, suggesting that a large proportion of the study participants did experience no or mild gut complaints, results that are in line with our previous study [4]. Thus, the study population was also comprised of persons experiencing no gastrointestinal symptoms, without the possibility to experience an improvement during the intervention. It is therefore likely that the outlined study would have gained from a larger sample size and/or a more homogenous population focusing on one particular gut problem. It is, however, important to consider that one can expect rather modest treatment effects of probiotic compounds. Thus, a population experiencing severe gut problems might not be responsive to a probiotic effect.

The enrolled study population was found to experience a better health status as judged by the low use of prescribed medications according to reports from the Swedish National Board of Health and Welfare [28]. It should be noted that self-reporting of medication in this age-group is problematic and often result in under-reporting [29]. However, in this study data regarding medication was collected in the home of the older adult with supported from the contact person.

Another aspect to consider in the RCT is the use of GSRS as primary outcome measure. An additional more fine tuned instrument such as the ROME III symptom criteria, specifically developed for irritable bowel syndrome (IBS), might have increased the sensitivity and allowed for detection of more subtle changes in gastrointestinal health throughout the study. In addition, it is a risk that subjects suffering from IBS were not identified and monitored throughout the study. However, it should be noted that IBS patients responding positive to ROME III symptom criteria commonly display more severe symptoms as measured by GSRS modified for IBS [30]. Thus, it is likely that participants suffering from IBS were included in the study, but, nevertheless, it is a limitation that these subjects could not be specifically identified, as that would have been an important finding and allowed for sub analyses.

Another reason for the negative outcome might be a too low dose of *L. reuteri*. The dose was identical to a previous clinical trial, reporting positive effects of *L. reuteri* on constipation in adults [15]. However, *L. reuteri* has been found to be more prevalent in subjects over 66 years of age [31]. Thus, suggesting that a higher dose might be needed in order to obtain a positive effect in older adults.

In addition, an inadequate number of study participants were enrolled. Five hundred eleven older adults in the region of Örebro responded to our advertisement and notified their interest to participate in the study. Three hundred and seven was still interested to participate after receiving an information letter regarding the study. However, given the low number of participants that were included in the final analysis (*n* = 249), additional recruitment strategies should have been applied. Thus, the study was underpowered. In addition, we made the decision to exclude 71 subjects from the final analysis as PPI and GMMS was found to affect and interfere with the primary outcome parameter.

Use of PPI have been found to be associated with enteric *Clostridium difficile* infection (CDI) [32]. CDI is commonly associated with diarrhea and long-term use of antibiotics [33]. Thus, PPIs have the potential to affect the composition of the gut microbiota and stool frequency. In the current study antibiotic use the last six months prior study start was an exclusion criterion. It is therefore likely that no subject suffered from *Clostridium difficile* associated diarrhea. Nevertheless, given the potential of PPI to affect the gastrointestinal tract use of PPI should have been an exclusion criterion, particularly as we were not able to recruit a sample size that allowed for sub analysis.

GMMS is frequently used in the treatment of constipation and diarrhea among older adults, regulating the intestinal motility and stool frequency. Clearly, it would have been a benefit for the study to set use of GMMS as an exclusion criterion. However, in a pilot study it was observed that older adults declined participation when asked not to take GMMS during a month. Hence, we decided to analyze the effect of GMMS intake in subsequent sub analysis after study finish.

In addition, 33 participants had to be excluded due to missing or incomplete CRF. It is possible that data from these participants would have an impact on the results. However, we would still not reach the minimal required sample size (*n* = 150) per arm. In addition, 28 questionnaires (HADS: 11; PSS: 17), had to be excluded from the analysis due to missing values. For both PSS and HADS no more than one item can be missed in order for the questionnaire to be included in the analysis. Given that 249 participants were included in the final analysis the excluded questionnaires represent a modest proportion and complete data sets would probably not affect the outcome for the secondary parameters radically. It is interesting to note that no items were missed when the study participants filled out GSRS, the primary outcome measure. Further, emphasizing that this instrument is easy and works well in an older population. All participants did fulfill the inclusion criteria and were mentally fit to complete the questionnaires. Only eight participants scored below 26 on the Mini mental state exam (MMSE), indicating mild cognitive impairment (data not shown). However, mild cognitive impairment rarely causes changes severe enough to interfere with daily life or independent function and probably did not affect their ability to complete the questionnaires.

Even though the trial had a negative outcome on the primary parameter we did observe a modest effect of probiotic treatment on stress level at week 8 and 12. Similarly, we found that subjects suffering from indigestion and abdominal pain, respectively, showed a significant decrease of anxiety at week 8 after probiotic treatment, but not at week 12. Similar findings have previously been reported and probiotic treatment has been found to have an influence on anxiety, as measured by HADS [34]. It should, however, be noted that our results are based on self-reported data and thus, rely on the respondents’ honesty, accurate understanding and interpretation on the questions asked. Furthermore, it is important to point out that the findings presented here have not been corrected for multiple comparisons, thus the *p*-values shown are only descriptive and should be interpreted with caution.

In addition, the study product used in the study also contained galactooligosaccharide a well-known prebiotic fiber, known to have a positive impact on the gut microbiota [35]. Thus, we cannot exclude that the modest effects observed in the trial is due to a synbiotic effect.

Despite the negative outcome, the results outlined in the present study highlights important issues that needs to be taken into consideration when designing clinical trials in older adults, particularly the high drop out rate in this part of the population is essential to reflect upon before starting the recruitment process.

In addition, it is evident that promoting gut health is important in order to increase the over all health status among older adults. Further studies will have to thoroughly investigate the gut-brain axis and how this signaling pathway changes with age. It will particularly be important to investigate the relationship between common gut problems, such as constipation and diarrhea, and the composition of the gut microbiota in older adults. This will gain insight on how aging affects gastrointestinal function and will be essential knowledge to build on when designing new RCTs to elucidate the effect of pro- and prebiotic compounds in older adults.

## Conclusion

The RCT failed to show any improvement in digestive health after daily intake of a supplement containing the probiotic strain *L. reuteri* during a three-month period. Neither was any significant improvement in wellbeing, stress or anxiety observed. Even though the trial had a negative outcome, the study highlights important issues that need to be taken into consideration when designing clinical trials in older adults. Furthermore, it is evident that promoting gut health may impact the over all health status, including mental health, among older adults.
